# Placebo Effect in the Treatment of Patellar Tendinopathy and Its Influencing Factors: Systematic Review With Meta-analysis and Meta Regression of Randomized Controlled Trials

**DOI:** 10.1177/23259671241258477

**Published:** 2024-08-28

**Authors:** Davide Previtali, Jacopo Albanese, Iacopo Romandini, Giulia Merli, Francesca Taraballi, Giuseppe Filardo

**Affiliations:** * Service of Orthopaedics and Traumatology, Department of Surgery, EOC, Lugano, Switzerland; † Department of Orthopaedics and Trauma Surgery, Kantonsspital, Saint Gallen, Switzerland; ‡ II Clinica, IRCCS Istituto Ortopedico Rizzoli, Bologna, Italy; § Applied and Translational Research (ATR) Center, IRCCS Istituto Ortopedico Rizzoli, Bologna, Italy; ¶ Center for Musculoskeletal Regeneration, Houston Methodist Research Institute, Houston, Texas, USA; # Orthopedics and Sports Medicine, Houston Methodist Hospital, Houston, Texas, USA; ** Faculty of Biomedical Sciences, Università della Svizzera Italiana, Lugano, Switzerland; Investigation performed at Ospedale Regionale di Lugano, EOC, Lugano, Switzerland and IRCCS Istituto Ortopedico Rizzoli, Bologna, Italy

**Keywords:** patellar tendinopathy, placebo effect, extracorporeal shock wave therapy, injections, taping

## Abstract

**Background:**

The effectiveness of nonsurgical treatment of patellar tendinopathy is questioned due to the conflicting results of placebo-controlled randomized controlled trials (RCTs) in which placebo arms often show impressive results.

**Purpose:**

To quantify the magnitude of placebo effect of the different nonsurgical treatments of patellar tendinopathy. We also evaluated the influence of patients and treatments characteristics on the response to the placebo.

**Study Design:**

Systematic review; Level of evidence, 1.

**Methods:**

We searched PubMed, Web of Science, Embase, Scopus, Cochrane Library, and gray literature databases on May 10, 2023, with no time limitation. RCTs on nonoperative treatment of patellar tendinopathy, including a placebo control arm reporting the evolution of symptoms after placebo administration, were included. A single-arm meta-analysis was performed with the Victorian Institute of Sport Assessment-Patella (VISA-P) at mid-term follow-up (3-6 months) as the primary outcome. The VISA-P score at short-term (1-3 months) and long-term (6-12 months) follow-ups, as well as visual analog scale (VAS) for pain at all 3 time points were also analyzed. A subanalysis based on the type of placebo and a meta-regression were conducted to look for potential determinants of the placebo effect. Risk of bias and level of evidence were also analyzed using the revised tool for risk of bias in randomized trials and Grading of Recommendations Assessment, Development and Evaluation.

**Results:**

In total, 14 studies (251 patients) were included. VISA-P score at mid-term follow-up (3-6 months) showed statistically significant improvements of 13 of 100 points (*P* = .001). The change at short-term follow-up (1-3 months) was not statistically significant, whereas at long-term follow-up (6-12 months) it was 27 of 100 points (*P <* .001). Regarding VAS, results were statistically significant only at mid-term (MD = −1.5/10; *P* = .02) and long-term (MD = −3.2/10; *P <* .001) follow-ups. The meta-regression showed positive correlations between the response to placebo and the follow-up length (*P* < .001) and the effect size in the experimental group (*P* = .006). The level of evidence was moderate for mid- and long-term results and low for short-term results.

**Conclusion:**

The placebo effect for nonsurgical treatments of patellar tendinopathy is long-lasting (up to 12 months) and statistically and clinically significant. It has a perceived and true component and differs among treatments. The duration of follow-up and the effect size of experimental groups correlate with the magnitude of the placebo component, underlining the importance of RCTs to determine the effectiveness of new treatments of patellar tendinopathy.

Patellar tendinopathy, or jumper’s knee, is a common sports-related disease with a prevalence ranging from 8.5% among amateur athletes up to 45% in professional volleyball players.^34,65^ The cause of the disorder seems to be multifactorial,^15,16,34^ and several factors, such as activity exposure hours, body mass index (BMI), age, and sex, have been considered possible determinants. Besides these factors, patellar tendon overuse during repeated jumps, with a paradoxical adaptation to load, is considered the main trigger of the tendinosis that characterizes the affected tissue.^9,38^ The main features of the tendinopathy are the progressive “mucoid” degeneration of the normal tendon with insufficient reparative and turnover capacity together with the presence of abnormal growth factor and gene expression, inflammatory mediators, and insufficient neovascularization.^19,24,30^ Moreover, the development of pain sensitization has been considered among the possible determinants of symptoms.^41^

To tackle the pathophysiological processes that sustain patellar tendinopathy with the aim of reducing its symptoms, various treatments have been developed such as exercise protocols, extracorporeal shock wave therapy (ESWT), ultrasonography, taping, and injections.^5,11^ Nonetheless, the current evidence on the effectiveness of the available solutions is weak and conflicting.^13,53^ Although apparently effective in single-arm studies, they often fail to confirm the early promising results in placebo-controlled randomized clinical trials (RCTs).^5,15^ One of the main challenges for the experimental treatment in RCTs on patellar tendinopathy is to overcome the placebo effect, which is often strong.^27,42^ The response to placebo treatments can be due to several factors, related to either a perceived or true placebo effect.^10,40,47^ In this light, quantifying the placebo component of the different treatments of patellar tendinopathy and identifying its related factors are keys to both properly designing scientific studies and better treating patients in clinical practice.

The main aim of this RCT meta-analysis was to quantify the placebo effect on pain and on the functional outcomes in the field of patellar tendinopathy. A meta-regression and subanalyses have been conducted to highlight possible discrepancies in the magnitude of placebo effect based on the characteristics of patients and symptoms or on the type of therapy administered.

## Methods

### Data Source and Study Selection Process

The systematic review of the literature was conducted following the PRISMA and PERSiST guidelines.^2,37^ After registration on PROSPERO (CRD4202344329), PubMed, Web of Science, Embase, Scopus, Cochrane Library, and gray literature databases were systematically searched on May 10, 2023, for eligible articles without time and language limitations using the following string: (patellar) AND ((tendinopathy) OR (tendinitis) OR (tendon pathology)) AND (saline OR placebo OR RCT OR randomized trial OR control arm). After removing duplicates, titles and abstracts were checked to exclude articles that were not relevant to the study purpose. The references of the included studies were further examined to retrieve other potentially includible trials. The article-selection process was performed independently by 2 authors (I.R., J.A.), with disagreement on study eligibility handled and solved by a third author (D.P.).

### Inclusion and Exclusion Criteria

The inclusion criteria were as follows: published RCTs (levels 1 or 2) on patellar tendinopathy including a placebo (as defined by the study authors) control arm; studies reporting both baseline and follow-up data (or their difference) for at least 1 outcome; and human studies. Studies that did not focus on patellar tendinopathy, nonrandomized placebo-controlled trials, unpublished manuscripts, preclinical studies, and conference abstracts were excluded.

### Data Extraction

The baseline data items that were collected included the following: level of evidence; study design; inclusion/exclusion criteria; blinding procedure; randomization procedure; follow-up length; and description of experimental and placebo treatments. Moreover, the following data on the study population were extracted: number of patients screened, included, and lost to follow-up; sex; age; BMI; patellar tendinopathy grade; patient-reported outcome measures; functional outcomes; knee range of motion; responder rate; complication rate; and radiological results. When standard deviations were not available from the full-text articles, they were computed using established methods.^22,26^ When results were not reported in text or tables but were available in graphs, data were electronically extracted from the graphs following the Cochrane guidelines.^29,60^ The data extraction process was performed independently by 2 authors (I.R., J.A.) and finally checked by a third author (D.P.).

### Outcomes and Measures

The prespecified primary outcome of the study was the Victorian Institute for Sport Assessment- Patella (VISA-P) score at midterm follow-up (3-6 months). The VISA-P was chosen as the primary outcome since it is the most used functional score in the evaluation of patellar tendinopathy.^59^ The midterm follow-up was chosen as the primary outcome since it is considered the minimum follow-up needed before proposing surgery in the absence of response.^31^ Secondary outcomes of the meta-analysis were the VISA-P at short-term (*<*3 months) and long-term (*>*6 months) follow-ups and visual analog scale (VAS) at short-term (*<*3 months), mid-term (3-6 months), and long-term (*>*6 months) follow-ups. The clinical relevance of the improvement was studied in terms of minimal clinically important difference (MCID), set at 1.2 of 10-point improvement for VAS and 13 of 100 points for VISA-P, as suggested by the literature.^6,21^ The other patient-reported outcome measures were reported in a low number of studies and thus could not be included in the meta-analysis. Regarding the meta-regression, to provide a more powerful analysis and avoid excluding studies reporting different patient-reported outcomes at different follow-ups (as would have happened if the subanalysis was based on specific patient-reported outcome measures and follow-up length), it was based on an overall meta-analysis including all the studies computed using the results of the longest follow-up reported in each study in terms of VISA-P (when reported), VAS (when VISA-P was not reported), or other scores (when both VISA-P and VAS were not reported). In the overall meta-analysis, an improvement on the VAS, which is normally represented by a negative change (since greater values represent worse pain), was reported as a positive value to make it comparable with an improvement on the VISA-P scale (in which greater values represent better results). Furthermore, when VAS was reported on a scale of 0 to 10, a simple proportion was computed to transform the scale of 0 to 10 scale to a scale of 0 to 100, making it comparable with the VISA-P, normally represented on a scale of 0 to 100. Subanalyses based on the type of placebo procedure used were performed. Finally, the influence on the placebo effect on type of procedure, sex, age, BMI, activity level, symptoms length, baseline pain, improvement in the experimental group, and length of follow-up were tested.

### Assessment of Risk of Bias and Quality of Evidence

The risk of bias was assessed using the revised tool for risk of bias in randomized trials approved by the Cochrane Collaboration group, which defines 3 categories: low risk of bias; some concerns; and high risk of bias.^54^ The overall quality of evidence for each outcome was graded according to the Grading of Recommendations Assessment, Development and Evaluation guidelines as high, moderate, low, and very low levels of evidence.^49^ The process was performed independently by 2 authors (I.R., J.A.) with disagreement handled and solved by a third author (D.P.).

### Statistical Analysis

The placebo effect was evaluated using a single-arm meta-analysis computing the results of the placebo arms of the included trials. For continuous variables, the placebo effect was expressed as the mean of the improvements from baseline to the different follow-ups. Dichotomous variables were not reported in enough trials to perform a meta-analysis. The random effect model was preferred to account for the heterogeneity of the included trials. An overall meta-analysis on VISA-P or VAS for pain (if VISA-P was not reported; conversion from a 0-10 scale to a 0-100 scale was made with a simple proportion), including the longest available follow-ups for each study, was computed to perform a meta-regression analysis. Separate linear meta-regressions were conducted to analyze possible influencing factors. In the meta-regression, the influence of the experimental treatment results was computed using the reported Cohen effect size of the experimental group of the included studies. A multiple meta-regression was not feasible due to the low number of included studies.^23^ A *P* value of .05 was set as the level of significance. To graphically estimate the impact of the placebo effect on the results of the experimental treatments of the included trials, we performed a single-arm meta-analysis computing the results at the last follow-up available of each experimental treatment in terms of VISA-P. We performed a statistical analysis with meta (v4.9-7, Schwarzer G, 2007) and metafor (v2.1-0, Viechtbauer W, 2010) packages in RStudio (v1.2.5019).

## Results

### Article Selection and Patients’ Characteristics

**Figure 1.**
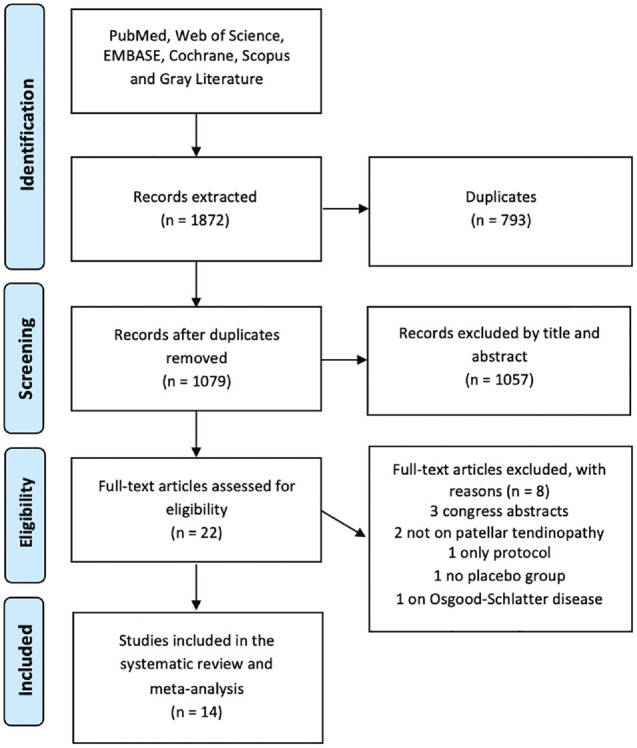
PRISMA (Preferred Reporting Items for Systematic Reviews and Meta-Analyses) flowchart of the study selection process.

The article selection process is shown in Figure 1. The database search retrieved 1872 articles, of which 14 were included in the meta-analysis.^††^ These studies were placebo-controlled RCTs focused on different therapeutic approaches for patellar tendinopathy: 5 studies were on intra-tendinous injections,^14,33,36,44,50^ 4 were on ESWT,^39,57,64,66^ 2 were on medicated patch,^45,55^ 1 on taping,^8^ 1 on low-intensity pulsed ultrasound,^61^ and 1 on pills.^48^ Regarding the blinding procedure, 11 studies were double-blinded (both patients and assessors),^‡‡^ 2 were single-blinded (patients),^33,50^ and 1 was not blinded.^8^ The mean age of the patients included in the placebo group was in the range of 23 to 34 years. All the studies included more men than women in the placebo group (except for the study by Sánchez-Gómez et al,^48^ which included the same number of men and women), the duration of symptoms varied between 8 and 40 months, and the activity level varied between 2.9 and 7.3 hours per week. More details on studies and patient characteristics are reported in Table 1.

**Table 1:** Characteristics of the Included Studies and Patients^*a*^

Study	Experimental Treatment	Inclusion and Diagnostic Criteria	Exclusion Criteria	Blinding	Type of Placebo	Patients Included	F-U Length (m)
de Vries et al,^8^ 2016	Taping	Age 18-50 y; patellar tendon pain and tenderness; symptoms length >3 m; VISA-P <80, active in sports	Acute patellar tendon problems; chronic joint diseases or other knee pathologies or surgery; inability to perform tests	No blinding	Placebo Taping	9 M, 7 F	0.3
Fredberg et al,^14^ 2004	Steroid injection	Symptomatic unilateral tendinitis at stage 3A/B >6 m, failed conservative treatment, US thickening (>1 mm)	Rupture of the tendon, steroids, infections, earlier surgery, diabetes, general inflammatory disease	Double-blind	3.5 mL 1% Lido + 0.5 mL intralipid injection	18 M, 6 F	1.0
Persson Krogh et al,^39^ 2021	ESWT (+ FKT)	Symptoms >6 m. At US swelling >1mm and central hypoechoic area >2 mm in diameter with hyperemia at Doppler	<18, >60 y, steroids or infections <6 m, previous surgery, rupture of the tendon	Double-blind	Placebo ESWT (+ FKT)	11 M, 7 F	3.0
Krogh et al,^33^ 2021	PRP (+ FKT)	Symptoms >6 m, failed rehab program. At US swelling >1mm	<18 y steroids or infections <6 m, previous surgery, inflammatory diseases	Single-blind (pts)	Saline injection (+ FKT)	10 M, 2 F	3.0
Olesen et al,^36^ 2021	IGF-1 (+ FKT)	18-50 y, 18-30 kg/m^2^ BMI, pain >3 m, tenderness. At US swelling >1mm with hyperemia at Doppler. 4 w without treatment	Previous surgery, osteoarthritis, pregnancy, diabetes, smoking, corticoid injection <12 m, contralateral PT	Double-blind	Saline injection (+ FKT)	15 M, 5 F	12.0
Resteghini et al,^44^ 2016	Autologous blood (+ FKT)	>18 y, PT with confirmed US diagnosis, failed conservative treatment and steroid injection	Previous allergic reaction to local anesthetic or steroid, infections, anticoagulants, vascular disease	Double-blind	Saline injection (+ FKT)	8 M, 3 F	12.0
Rigby et al,^45^ 2015	Iontophoresis	18-45 y, pain >1 m <2 y, clinical diagnosis of midsubstance PT	Contraindications to steroids or iontophoresis, surgery <6 m, confounding diagnoses steroids <2 m, painkillers, inflammation	Double-blind	Placebo Patch	10 M	0.5
Sánchez-Gómez et al,^48^ 2022	β-hHdroxy β-methylbutyric pills (+ FKT)	Athletes with PT, 18-49 y	Previous surgery or infiltration, substance that could affect their hormones or performance <3 m, allergy, smokers, other disorders	Double-blind	Placebo pills (+ FKT)	2 M, 2 F	1.0
Scott et al,^50^ 2019	PRP (+ FKT)	18-50 y, PT pain and tenderness >6 m, at US swelling and central hypoechoic area, failed rehab, normal hematology	Previous surgery, inflammatory diseases, osteoarthritis	Single-blind (pts)	Saline injection (+ FKT)	18 M, 3 F	12.0
Steunebrink et al,^55^ 2013	Glyceryl trinitrate patch	Chronic PT (>3 m), 18-40 y, pain or thickened tendon	Pain >24 m, VISA-P >80, previous surgery or injection, previous heavy-load eccentric exercise, pregnancy, GTN contraindication	Double-blind	Placebo Patch	14 M, 3 F	6.0
Thijs et al,^57^ 2017	ESWT (+ FKT)	Sports >1/w, 18-40 y, pain, tenderness > 8 w, VISA-P <80, no PF pain	Acute injury, other diseases, immunosuppression or steroids <6 m, previous surgery, injection <1 m, ESWT contraindications, previous ESWT	Double-blind	Placebo ESWT (+ FKT)	24 M, 6 F	6.0
Warden et al,^61^ 2008	LIPUS (+ FKT)	>18 y, pain, tenderness affecting exercise >6m, VISA-P <80, US hypoechoic lesion	Other diseases, previous surgery, injection <6 m	Double-blind	Placebo LIPUS (+ FKT)	18 M, 2 F	3.0
Zhang et al,^64^ 2020	ESWT	18-35 y, pain 3 m, VAS≥ 3, VISA-P <80, pain and tenderness, thickening and hypoechoic signals on US	Previous surgery or steroid injection	Double-blind	Placebo ESWT	17 M	Immediate
Zwerver et al,^66^ 2011	ESWT	Pain, tenderness for 3-12 m, 18-35 y, VISA-P <80	Acute knee injuries, other disorders, long-term NSAIDs, fluoroquinolones, previous surgery steroid injections <3 m, ESWT contraindications	Double-blind	Placebo ESWT	21 M, 10 F	6.0

a +FKT, physiotherapy advised during the study period; BMI, body mass index; ESWT, extra-corporeal shock-wave therapy; F, female; F-U, follow-up; GTN, glyceryl trinitrate patch; IGF-1, insulin growth factor 1; LIPUS, low-intensity pulsed ultrasound; M, male; m, month; PF, patellofemoral; PRP, platelet-rich plasma; PT, patellar tendinopathy; pts, Patients; US, ultrasound; VISA-P, Victorian Institute for Sport Assessment-Patella score.

### Placebo Effect in Patellar Tendinopathy

**Figure 2.**
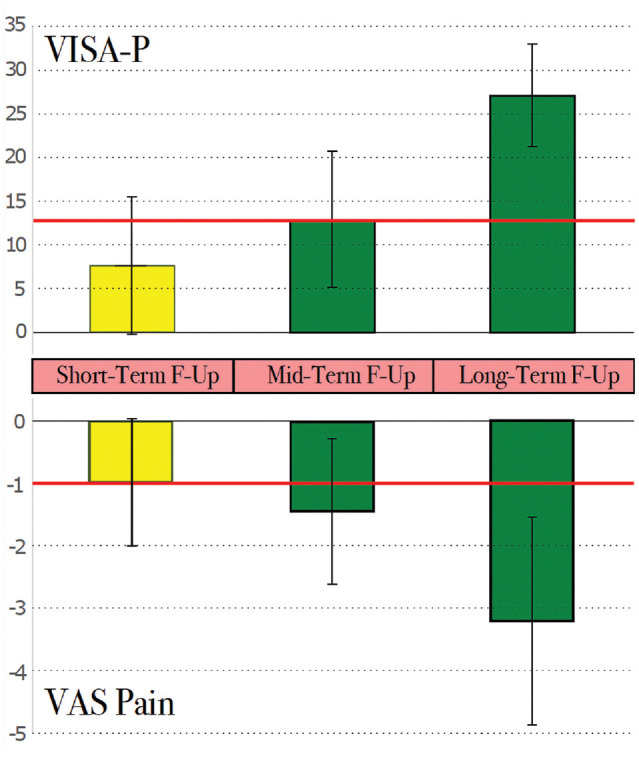
Box plot of the improvement after placebo administration in terms of VISA-P and VAS for pain 0-10 at short- (<3 months), mid- (3-6 months), and long-term (>6 months) follow-ups. Yellow indicates that the difference was not significant, whereas green indicates the difference was both statistically and clinically significant. The red line identifies the minimally clinical important difference. F-up, follow-up; VISA-P, Victorian Institute for Sport Assessment-Patella; VAS, visual analog scale.

The meta-analysis of the results of placebo in terms of VISA-P at the mid-term follow-up, the primary outcome of the meta-analysis, showed an overall statistically significant improvement of 13 of 100 points (range, 5-21; *P* = .001). The change from baseline at the short-term follow-up was not statistically significant at 7.8 of 100 points (range, −0.9 to 16.4; *P* = .08) whereas the change at the long-term follow-up was a statistically significant improvement of 27 of 100 points (range, 22-33; *P* < .001) (Figure 2).

Regarding VAS, the results were not statistically significant at the short-term follow-up (MD = −1/10; range, −2.0 to 0.1; *P* = .07) and statistically significant at both the mid-term (MD = −1.5/10; range, −2.6 to −0.3; *P* = .02) and long-term (MD = −3.2; range, −4.9 to −1.5; *P* < .001) follow-ups (Figure 2).

The overall meta-analysis at the longest available follow-up reported a statistically significant improvement of 11.3 of 100 points (range, 3.9-18.8; *P* = .003).

### Determinants of Placebo Effect

**Figure 3.**
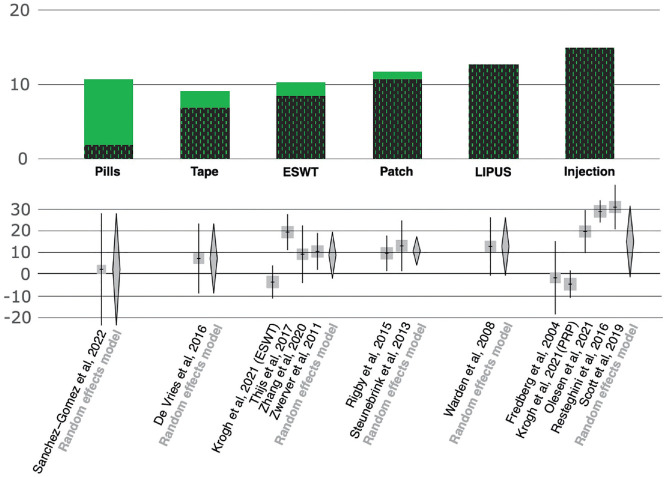
Bar plot and forest plots showing the magnitude of the placebo component of the different types of conservative treatment in terms of VISA-P at the last follow-up available for each study. The bar plot in the upper part of the figure shows in green the results of the meta-analyses of the different types of experimental treatments reported in the included randomized controlled trials (ie, response to experimental treatment), whereas the superimposed black dotted green bars represent the results of the meta-analyses of the different types of placebos (ie, the placebo component of the response). Below, the forest plots of placebo response in terms of VISA-P at the longest follow-up available with subanalyses based on the type of placebo are represented. ESWT, extracorporeal shock-wave therapy; LIPUS, low-intensity pulsed ultrasound; PRP, platelet-rich plasma; VISA-P, Victorian Institute for Sport Assessment-Patella.

The presence of possible determinants of the placebo effect was tested with a meta-regression. A statistically significant correlation was present for the length of follow-up (coefficient 2.1; *P* < .001) and the effect size in the experimental group (coefficient 8.0; *P* = 0.02) (Figure 3). No statistically significant correlation was found for the type of placebo, patient age, sex, BMI, hours of activity per week, symptoms length, and baseline symptoms intensity.

### Risk of Bias and Quality of Evidence

Of the included trials, 9 had a low risk of bias, whereas 4 presented some concerns of bias and 1 presented a high risk of bias. The absence of indications on the method used to maintain allocation concealment was the most common source of possible bias. The lack of physician blinding in 2 of the studies was not considered to be a possible source of bias due to the absence of unbalanced deviations from the original protocol due to group assignment. The lack of blinding of both physicians and patients implied a high risk of bias in the study by de Vries et al (additional details are reported in the supplementary material).^8^

The level of evidence for the presence of a statistically and clinically significant placebo effect in the treatment of patellar tendinopathy is moderate for the mid- and long-term follow-ups for both the VISA-P and VAS scores, and low for the short-term follow-up for both the VISA-P and VAS scores. The main reason for downgrading the level of evidence was inconsistency (high heterogeneity) in the results of the meta-analysis (additional details are reported in the supplementary material).

## Discussion

The main finding of this study is that nonsurgical treatments of patellar tendinopathy are characterized by the presence of a statistically and clinically significant placebo effect. A statistically significant improvement was documented, overcoming the MCID of 13 points previously reported for the VISA-P, in the mid- and long-term follow-up meta-analyses.^21^ Regarding VAS, the MCID for the nonsurgical treatment of patellar tendinopathy has recently been determined and set as a change of 1.2 of 10 points.^6^ The documented changes of 1.5 and 3.2 points at the mid- and long-term follow-ups, respectively, both overcame the MCID.

These findings, showing the magnitude and the persistence of the placebo effect in the nonsurgical treatment of patellar tendinopathy, underline the importance of placebo-controlled trials to establish the real effectiveness of an experimental treatment. An improvement documented for a hypothetically effective treatment, even if long-lasting and statistically and clinically significant, may be due only to the placebo effect.^43^ Recently, placebome research has allowed for the identification of a series of biological pathways that might identify good placebo responders.^17^ From a clinical perspective, predicting placebo responders could modify clinical approaches and optimize clinical care. Although we are still far from understanding the complex physiology of the placebo effect, we can attempt to understand and clarify the placebo effect from previous studies and define its clinical relevance.

The results of the subanalysis performed may help clarify the nature and identify the possible determinants of the placebo effect. Both the subanalysis based on the follow-up length and the meta-regression clearly showed that the length of follow-up influences the improvements in the placebo groups of the included RCTs. At longer follow-ups, a higher placebo effect is documented. An explanation of this finding may be sought in the nature of the placebo effects itself by distinguishing between the perceived and the true placebo-effect components. The perceived placebo effect is the result of various components and may go beyond the true placebo effect when the improvement of symptoms is not properly attributable to the placebo but rather to the characteristics of the disease or to the clinical trial in which this effect is recorded.^10^ In the case of patellar tendinopathy, the natural history of the disease and the regression to the mean phenomenon may be an important confounder in determining the magnitude of the placebo effect.^18^ Patellar tendinopathy, like other tendon diseases, is characterized by an irregular progression with improvements in symptoms and outbreaks that eventually leads to a symptom-free state after a medium to long period of activity restriction.^30^ In this light, the regression to the mean and the natural history of patellar tendinopathy causing a perceived placebo effect may lead to a misinterpretation of the results of the treatment of patellar tendinopathy.

These considerations are also consistent with other determinants of the placebo effect emerging from meta-regression, which is the response documented in the experimental group. The improvement unavoidably influences the results in the experimental group due to the natural history of the disease and the regression to the mean. At the same time, the tendency of patients included in clinical trials to modify their behaviors to more appropriate habits, coined the “Hawthorne effect,” may play a role in the perceived placebo effect and in the correlation between the results of experimental and control groups.^35^ Among others, the concomitant autonomous or suggested switch to appropriate exercise programs should always be considered in the evaluation of improvements after new experimental treatments.^58^ In at least 8 of 14 studies,^33,36,39,44,48,50,57,61^ an exercise protocol was followed by study participants alongside the evaluated treatment and had a probable influence on response. Similarly, the removal of potential modifiable risk factors may play a confounding role in the improvement documented after treatment causing a perceived placebo effect, even if their importance according to the literature is still not clear.^52^ In this regard, at least a part of the placebo effect perceived at the midterm and long-term follow-ups may be due to the natural evolution of patellar tendinopathy or to the characteristics of clinical trials and not to a true placebo effect.

To better understand the importance of the true placebo effect in patellar tendinopathy, a look should be given to potential indicators of the influence on its results of placebo determinants, such as conditioning, expectations, and meaning.^32,62^ In particular, the subanalysis based on the type of placebo could be useful to understand whether the administration modality, which has been documented to be relevant in the expectation of benefit in other musculoskeletal diseases, also plays a crucial role in patellar tendinopathy.^63^ In this regard, injected placebo seems to be more effective compared with other administration modalities, thus supporting the possible influence of a true placebo effect on the results. Moreover, a key finding is the statistically significant correlation between the effect size in the experimental group and the placebo effect. Accordingly, the supposed higher improvement observed with injected treatments, underlined by the most recent meta-analyses, may be due to a related higher placebo component of the injectable treatment approach opposed to a higher efficacy.^1,3,7^

The tendency of injections in provoking a stronger placebo effect also raises another important question about the placebo nature of the observed improvement. Placebo injections imply a dry needling; even the injections of saline cause hydrodissection and physical changes at the abnormal tissue level and around the patellar tendinopathy area.^51,56^ This approach is sometimes cited as an effective conservative procedure for patellar tendinopathy.^20,56^ In this light, at least for injections, the presence of a true therapeutic effect should also be accounted for as a possible determinant of the perceived (and not true) placebo effect. Thus, it could be debatable that RCTs comparing injectable approaches to a so-called “placebo” injective group are using an inert placebo or another treatment control group. In any case, regardless of the nature of the observed effects due to the perceived true placebo or real biological changes, new emerging injective treatments, including the fashionable orthobiologic approaches, should be tested in RCTs and prove superiority to the results offered by the more simple injection of saline, which can often provide a significant improvement accounting for most of the treatment benefit, as confirmed by this study.^50^

In addition to the problems identifying a real placebo for orthobiologic injections, this systematic review and meta-analysis of the literature indirectly highlight another important point in the scientific field of the conservative treatment of patellar tendinopathy. Even though several clinical trials are published every year claiming the effectiveness of a specific exercise protocol, the systematic review of the literature did not retrieve any study comparing a therapeutic exercise protocol to placebo exercise.^4,25,28,46^ This is most likely because it is not simple to define a “placebo exercise.” However, since the placebo effect in the conservative treatment of patellar tendinopathy is strong enough to provide a clinically significant improvement, placebo-controlled RCTs are paramount when evaluating the effectiveness of exercise protocols.

The meta-regression did not suggest other factors that may be useful in explaining the nature of the placebo effect in patellar tendinopathy. None of the patients and trial characteristics, such as sex, age, BMI, activity level, length of the disease, and baseline level of symptoms, reached statistical significance.

### Limitations

A limitation of our study is the lack of clinical significance regarding some of the performed analyses that could be due to the limited number of RCTs published on this topic and thus included in the analysis. Moreover, the short-term follow-up analysis included results from the first few days to the first 3 months, resulting in a heterogenous report that may have limited the possibility of finding significant results. The low number of studies also limited the possibility of performing a multiple meta-regression, which could provide stronger results. Another potential limitation of the meta-regression is that it had to be based on an overall meta-analysis computed using all the studies independently from the outcome used as described in the Methods section to improve its power. An additional limitation is the difficulty in accounting for factors such as the study context, physician attitude, and patient mood, which play a key role in determining the true placebo effect. The inclusion of nonblinded RCTs could also be considered a limitation, even if patients were not blinded in only 1 of the included studies.^8^ The unblinding of patients unavoidably influences the possibility of obtaining a true placebo effect and could even lead to a nocebo effect.^12^ Accordingly, this RCT does not document a significant improvement in the placebo group and is considered to have a high risk of bias. Lastly, even though most of the studies included were double-blinded RCTs with a low risk of bias, the high heterogeneity of the included trials and of their results, which has been only partially accounted for with subanalyses and meta-regression, represents a possible limitation.

Beside these limitations, our meta-analysis showed that for the nonsurgical treatment of patellar tendinopathy the placebo effect, both true and perceived, is long-lasting as well as statistically and clinically significant. It varies among the different treatments, being stronger for injections. This underlines the importance of double-blinded RCTs to determine the effectiveness of new treatments. To avoid misleading conclusions, possible determinants of a perceived and true placebo effect should always be accounted for in the clinical and research fields of nonsurgical treatment of patellar tendinopathy.

## Conclusion

The placebo effect for nonsurgical treatments of patellar tendinopathy is long-lasting (up to 12 months) and is statistically and clinically significant. It has a perceived and true component and differs among treatments. The duration of follow-up and the effect size of experimental groups correlate with the magnitude of the placebo component, underlining the importance of RCTs to determine the effectiveness of new treatments of patellar tendinopathy.

## Supplemental Material

sj-.xlsx-1-ojs-10.1177_23259671241258477 – Supplemental material for Placebo Effect in the Treatment of Patellar Tendinopathy and Its Influencing Factors: Systematic Review With Meta-analysis and Meta Regression of Randomized Controlled Trials Supplemental material, sj-.xlsx-1-ojs-10.1177_23259671241258477 for Placebo Effect in the Treatment of Patellar Tendinopathy and Its Influencing Factors: Systematic Review With Meta-analysis and Meta Regression of Randomized Controlled Trials by Davide Previtali, Jacopo Albanese, Iacopo Romandini, Giulia Merli, Francesca Taraballi and Giuseppe Filardo in Orthopaedic Journal of Sports Medicine

## Footnotes

†† References 8, 14, 33, 36, 39, 44, 45, 48, 50, 55, 57, 61, 64, 66.

‡‡ References 14, 36, 39, 44, 45, 48, 55, 57, 61, 64, 66.
